# RNA-protein complexes as key regulators of plant secondary metabolism: Biophysical interactions and functional significance

**DOI:** 10.1016/j.bbrep.2026.102664

**Published:** 2026-06-08

**Authors:** Okechukwu Paul-Chima Ugwu, Melvin Nnaemeka Ugwu, Hope Onohuean, Hilal Ahmad Rather, Ibe Michael Usman

**Affiliations:** a Department of Research, Publication and Extension, Kampala International University, Kampala, Uganda; b Department of Medical Biochemistry, Faculty of Basic Medical Sciences, State University of Medical and Applied Science, Igbo Eno, Enugu, Nigeria; c Biopharmaceutics Unit, Department of Pharmacology and Toxicology, School of Pharmacy, Kampala International University Uganda, Western Campus, Ishaka-Bushenyi, Uganda; d Department of Biochemistry Kampala International University Uganda, Western Campus, Uganda; e Department of Human Anatomy Faculty of Biomedical Science Kampala International University, Uganda

**Keywords:** RNA-Protein complexes, RNA-binding proteins, Secondary (specialised) metabolism, Stress granules, and liquid-liquid phase separation

## Abstract

RNA-protein complexes (RNPs) are central regulators of post-transcriptional gene expression and are increasingly implicated in controlling when, where and to what extent specialised (secondary) metabolic pathways operate in plants. Relative to transcription factor-centred regulation, RNPs can modify pathway output rapidly by modulating mRNA processing, stability, localisation and translation, thereby enabling plants to adjust metabolite production on developmentally and environmentally relevant timescales. Many regulatory RNPs assemble into membraneless biomolecular condensates (e.g., stress granules and processing bodies) via liquid-liquid phase separation (LLPS), providing reversible mechanisms to sequester, protect or degrade transcripts, including those encoding enzymes and regulators of specialised metabolism. In addition, small RNA-guided silencing complexes (e.g., AGO-containing RISC) form well-defined RNP modules that post-transcriptionally regulate transcription factors and biosynthetic genes involved in phenylpropanoid/flavonoid and other pathways. Here, we synthesise current evidence linking RNP biology to specialised metabolism, explicitly distinguishing mechanistic evidence (e.g., direct binding with functional perturbation) from associative evidence (e.g., co-localisation or proteomic enrichment). We also summarise experimental and computational approaches for mapping plant RNA-protein interactions, including key limitations (crosslinking bias, antibody specificity and contamination) and emerging tools (enhanced RNA interactome capture, proximity labelling and deep-learning-assisted binding prediction). Finally, we highlight evidence gaps in non-model medicinal plants and propose a framework that integrates spatiotemporal transcriptomics, structural biology and RNP-centred engineering to improve plant biofactories and stress-resilient phytochemical production.

## Highlights

• RNPs regulate specialised metabolism through post-transcriptional control of pathway RNAs.
• LLPS drives stress granules and P-bodies that store, repress or degrade mRNAs.
• RBPs bind mRNAs and lncRNAs/miRNAs/circRNAs to modulate biosynthetic gene output.
• RNP-mediated control can be faster and more reversible than transcription-factor regulation.
• Emerging tools map plant RNA-protein networks for metabolic engineering.

## Graphical abstract

**Figure:**
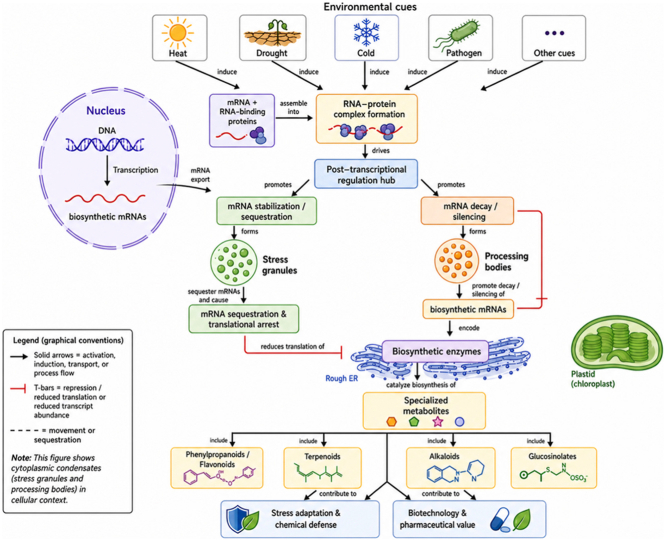

## 1 Introduction

**Fig. 1:**
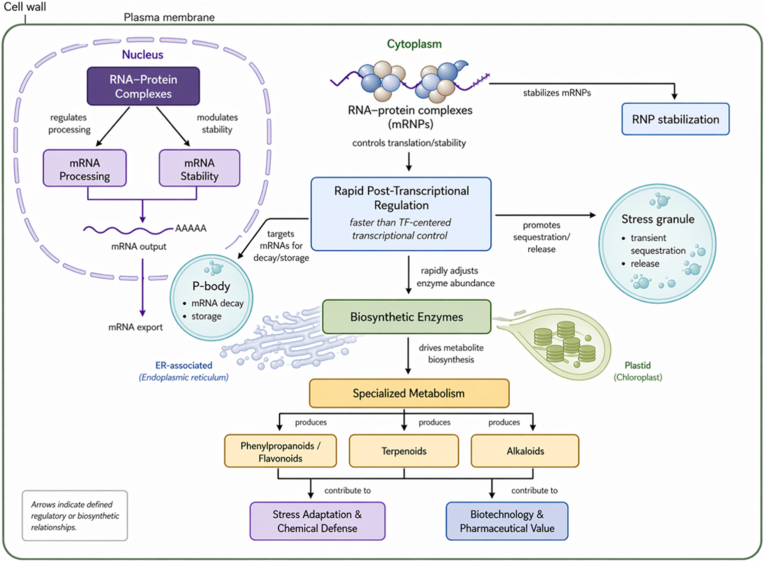
Post-transcriptional (RNP-mediated) control of plant specialised metabolism and metabolite output.

**Fig. 2:**
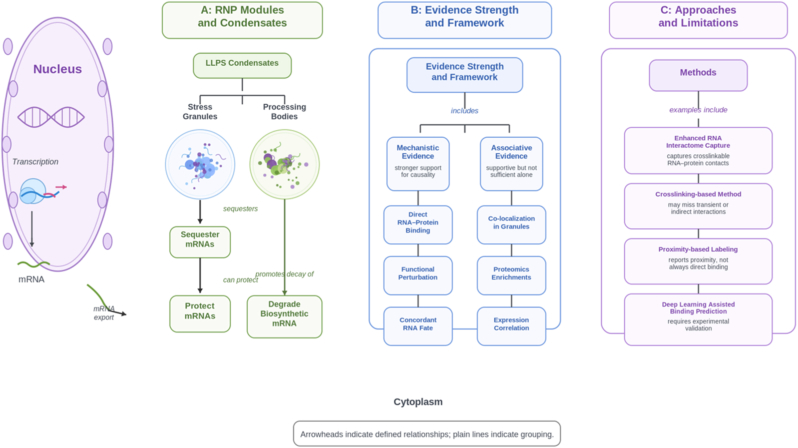
**RNP modules, evidence standards, and experimental approaches for linking RNA regulation to specialised metabolism (A) RNP modules and condensates:** RNA-protein (RNP) regulation occurs within liquid-liquid phase separation (LLPS) condensates, including stress granules and processing bodies, which can sequester transcripts and bias mRNA fate towards protection, storage, degradation, or regulated translation, thereby affecting biosynthetic mRNAs. In parallel, small RNA-guided silencing complexes (AGO-RISC) use a guide small RNA to recognise target mRNAs, causing translational repression or cleavage. These complexes often target transcription factors and biosynthetic genes across pathways, including phenylpropanoid/flavonoid (and others). **(B) Evidence strength framework:** Mechanistic evidence includes direct RNA-protein binding (e.g., CLIP/EMSA), functional perturbation (LOF/GOF), and concordant RNA fate and/or metabolite output. Associative evidence includes co-localisation in granules, proteomics enrichment, and expression correlation, including predictions that lack functional validation. **(C) Approaches and limitations:** Key methods include enhanced RNA interactome capture, crosslinking-based mapping (CLIP-seq family), proximity labelling (APEX/TurboID), and deep-learning-assisted binding prediction. Common caveats include crosslinking bias, antibody specificity issues, and contamination (Fig. 2).

Plants produce a wide range of specialised metabolites, including phenylpropanoids/flavonoids, terpenoids, alkaloids, and glucosinolates, which contribute to stress adaptation and chemical communication and provide substantial value for biotechnology and therapeutics (Fig. 1, Fig. 2) [1]. Because these pathways are energetically demanding and tightly coupled to cellular resource allocation, their outputs are regulated with marked spatial and temporal specificity across tissues, cell types, and environmental conditions [2]. Although transcription factor networks and chromatin state are central determinants of biosynthetic gene expression [3], transcriptional control alone often cannot explain the speed, reversibility, and subcellular specificity of metabolic reprogramming, particularly when plants adjust pathway flux over minutes to hours [[4], [5], [6]].

**Fig. 3:**
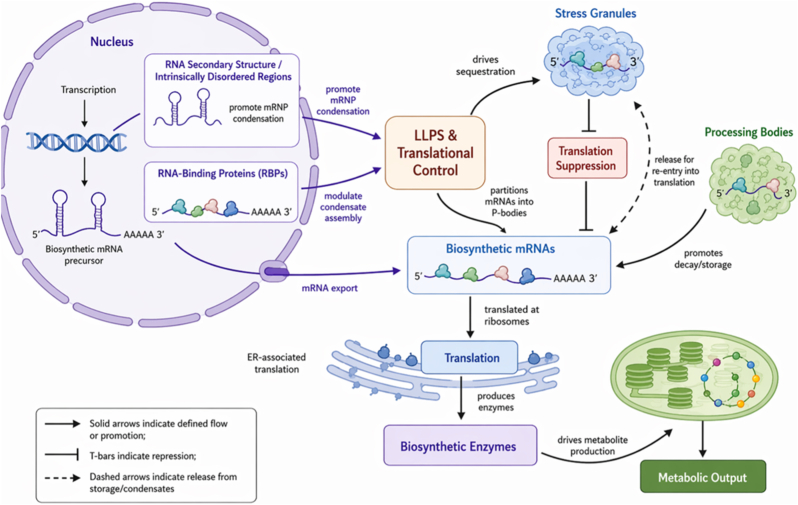
**Stress-and LLPS-driven RNP condensates reprogramme translation of biosynthetic mRNAs in plant cells** Schematic suggesting how stress cues promote liquid-liquid phase separation (LLPS) in the plant cytoplasm, driving assembly of messenger ribonucleoprotein (mRNP) condensates, including stress granules and processing bodies. RNA-binding proteins (RBPs), which are often enriched in intrinsically disordered regions, together with RNA secondary-structure features, facilitate RNP condensation and selective recruitment of mRNAs. These condensates modulate translational control by transiently storing, repressing, or directing transcripts between translation-competent pools and decay-associated pathways. Consequently, translation of biosynthetic mRNAs is dynamically tuned, altering biosynthetic enzyme abundance and downstream specialised-metabolite output (Fig. 3).

Post-transcriptional regulation provides a complementary control layer in which ribonucleoprotein complexes (RNPs) determine the fate of pathway-relevant RNAs after transcription [5]. Here, ‘RNP’ denotes functional assemblies of RNA with RNA-binding proteins (RBPs) that modulate RNA processing, localisation, stability, and translation, thereby tuning enzyme abundance and metabolic output independently of transcriptional initiation [[7], [8], [9], [10]]. This definition encompasses: (i) messenger RNPs that coordinate splicing, export, localisation, translation efficiency, and decay; (ii) dynamic RNP condensates, such as stress granules and processing bodies, that partition transcripts into storage, repression, or turnover; (iii) small RNA effector complexes, including AGO-containing RNA-induced silencing complexes (RISC), that execute sequence-directed repression or cleavage of transcripts encoding biosynthetic enzymes and upstream regulators; and (iv) organelle-associated RNP systems that indirectly influence specialised metabolism via redox balance, energy state, and precursor availability [[11], [12], [13]].

To improve interpretability, we distinguish mechanistic RBP-RNA regulation from associative signals using an explicit evidence-strength framework. Mechanistic evidence requires direct binding together with functional perturbation that yields concordant effects on RNA fate and/or metabolite output, whereas associative evidence includes co-localisation, enrichment, correlation, or prediction without functional validation. This structure enables a clearer synthesis of validated biochemical and biophysical mechanisms and highlights key evidence gaps, particularly in non-model and medicinal plants relevant to RNP-centred metabolic engineering.

Schematic overview of how RNA-protein complexes (RNPs) fine-tune specialised metabolism by regulating multiple mRNA fate checkpoints. RNPs influence mRNA processing (splicing/editing) and mRNA stability (protection versus decay), and coordinate mRNA localisation (subcellular targeting) and mRNA translation (protein output). These post-transcriptional controls determine the abundance and timing of biosynthetic enzymes, thereby shaping pathway flux and metabolite output. The specialised metabolite classes highlighted include phenylpropanoids/flavonoids, terpenoids, alkaloids, and glucosinolates, which contribute to stress adaptation, chemical communication, and biotechnology value. Arrows indicate regulatory flow from mRNA fate decisions to enzyme production and downstream metabolite output, highlighting rapid regulation alongside transcription factor (TF)-centred control (Fig. 1).

## 2 Methodology

This narrative review examines how plant ribonucleoprotein (RNP) biology contributes to post-transcriptional control of specialised metabolism, with emphasis on RNA fate decisions mRNA processing, stability, localisation, and translation that modulate biosynthetic enzyme abundance and metabolite output. The review is organised around predefined thematic modules (biomolecular condensates such as stress granules and processing bodies, small RNA-guided silencing pathways, and RNA-protein interaction networks) to provide a coherent mechanistic framework linking molecular regulation to pathway-level outcomes across phenylpropanoid/flavonoid, terpenoid, alkaloid, and other specialised metabolic systems, including applications relevant to medicinal plants.

A structured literature search was conducted in PubMed, Scopus, and Web of Science, prioritising peer-reviewed studies published from 2015 to 2025 (last searched: 16 December 2025). Search strategies combined keywords and controlled vocabulary, where available, using Boolean operators. Representative search strings included (‘RNA-binding protein*’ OR ribonucleoprotein* OR RNP) AND (‘stress granule*’ OR ‘processing bod*’ OR P-body* OR phase separation OR LLPS) AND (‘specialised metabolism’ OR ‘secondary metabolism’ OR flavonoid* OR phenylpropanoid* OR terpenoid* OR alkaloid* OR glucosinolate* OR ‘medicinal plant*’), with analogous combinations for RNA silencing and RNA-protein interaction methods (e.g., CLIP, interactome capture). Full search strings and database-specific syntax are provided in the Supplementary Methods. To improve coverage, we conducted backward reference screening of key papers and recent reviews, and forward citation tracking of foundational studies.

Eligibility was restricted to English-language, peer-reviewed primary research and authoritative syntheses in plant systems; conference abstracts, editorials, and non-peer-reviewed preprints were excluded. Non-plant studies were included only when they provided directly transferable mechanistic insight (e.g., conserved RNP principles or method benchmarks) and were explicitly labelled as contextual. Study selection and synthesis followed predefined relevance and evidence-strength criteria aligned with the review objective. Two reviewers independently screened titles and abstracts using a priori inclusion criteria (a plant RNP component or process with mechanistic or testable relevance to specialised metabolism), followed by full-text assessment; disagreements were resolved by discussion and consensus.

For each included study, key elements were charted in a standardised extraction table (RNP factor/complex, organism/tissue and experimental context, methods used, evidence for direct RNA-protein interaction or RNA fate modulation, RNA targets where available, and reported enzyme/metabolite outcomes). Evidence was appraised using an explicit hierarchy. Strongest weight was assigned to studies demonstrating: (i) direct RNA-protein binding (e.g., CLIP-family assays, EMSA); (ii) functional perturbation (loss- or gain-of-function) with phenotypic, biochemical, and/or metabolomic readouts; and (iii) concordant changes in RNA fate and metabolite output consistent with the proposed mechanism. Evidence based primarily on association such as co-localisation in granules, proteomics enrichment, expression correlation, or computational prediction without functional validation was retained to map emerging hypotheses and knowledge gaps but was explicitly labelled as associative and was not used alone to support causal claims. Method-specific limitations (including crosslinking bias, antibody specificity, and contamination in proximity-based workflows) were considered when interpreting inference strength; where orthogonal validation was lacking, conclusions were downgraded accordingly. Conflicting findings were summarized in parallel, with attention to differences in species, tissues, stress context, and assay design, to support balanced interpretation. As a narrative review, this synthesis does not follow a formal systematic-review protocol and does not include meta-analysis; conclusions therefore reflect thematic integration and transparent, criteria-based weighting of evidence rather than quantitative effect estimation.

### 2.1 RNA protein complexes in plant cells: A functional overview

In plant cells, RNA-protein complexes (RNPs) are versatile molecular assemblies that modulate gene expression post-transcription, as illustrated in Table 1 [14]. RNPs govern RNA fate across the RNA life cycle. By definition, messenger ribonucleoproteins (mRNPs) comprise mRNA and RNA-binding proteins (RBPs); they form co-transcriptionally and are remodelled during splicing, export, translation, and decay. These assemblies, which include one or more RNA molecules and multiple RBPs, function at nearly every stage of RNA processing [15]. RNPs can be classified according to composition, subcellular localisation, and functional specialisation [16]. mRNPs are extensively studied because they associate with messenger RNAs (mRNAs) throughout their life cycle and facilitate splicing, nuclear export, localisation, translation, and eventual degradation [17]. Collectively, these complexes support accurate mRNA processing and maintenance and thereby regulate gene expression [18].

**Table 1:** Representative ribonucleoprotein (RNP) modules and RNA-binding proteins (RBPs) with relevance to specialised metabolism (direct or regulatory) in plants.

RNP/RBP (Module)	Species	Compartment	Organ/tissue context	Main RNA target(s)/partner	Specialised-metabolism link	Evidence type	Citation/evidence
AGO1-containing RISC (miRNA effector RNP)	*Arabidopsis thaliana* (and widely conserved in plants)	Cytoplasm and nucleus	Leaves/various tissues	miRNA-mRNA pairing (TFs and enzymes)	Post-transcriptional regulation of flavonoid/phenylpropanoid regulators via miRNAs (e.g., MYB-related networks)	Mechanistic for miRNA cleavage/silencing as a process; pathway-specific links vary by miRNA/target	Reviews summarizing miRNA control of flavonoid/phenylpropanoid regulation [[14], [15], [16], [17], [18]]
DCL1–HYL1/DRB1 microprocessor (miRNA biogenesis RNP)	*A. thaliana*	Nucleus	Broad	pri-miRNAs to mature miRNAs	Enables miRNA-mediated tuning of specialised-metabolism networks	Mechanistic for miRNA biogenesis; pathway link depends on downstream miRNA targets	Plant dsRNA-binding proteins in RNAi-like pathways [[15], [16], [17], [18], [19]]
Stress granules (SGs; liquid–liquid phase-separated mRNP condensates)	*A. thaliana* and other plants	Cytoplasm	Heat, drought, and oxidative-stress contexts	Translationally stalled mRNAs plus RBPs	Rapid translational reprioritization during stress, plausibly affecting translation of defense-metabolite enzymes; often associative unless metabolite output is directly tested	Mainly associative for pathway links	Plant polysome, SG, and P-body dynamics overview [[17], [18], [19], [20], [21], [22], [23]]
Processing bodies (P-bodies)	*A. thaliana* and other plants	Cytoplasm	Broad; stress responsive	mRNA decay/repression factors plus mRNAs	Controls transcript turnover and can reshape availability of transcripts feeding defense programs	Associative to mechanistic, depending on target validation	Plant SG/P-body composition and function [24,25]
Organelle RBPs (e.g., PPR family; chloroplast/mitochondria RNA metabolism)	*A. thaliana* and crop species	Chloroplasts/mitochondria	Green tissues and organelle-rich cells	Organelle mRNAs (editing, splicing, stability)	Indirect effects through plastid metabolism, redox balance, and precursor supply feeding terpenoid/isoprenoid-related processes	Mechanistic for RNA metabolism; specialised-metabolism link often indirect	Organelle RNA metabolism and applications [26,27]
RBP-binding motif resources (ATtRACT, RBPDB, POSTAR3, and oRNAment)	Multi-species	In silico	Not applicable	Motifs, binding sites, and CLIP-supported interaction data	Supports hypothesis generation for plant RBPs and candidate RNA targets	Resource-level evidence	Representative resource/database papers and related reviews [[25], [26], [27], [28], [29], [30]]

This table presents representative RNP assemblies and RBPs implicated in the regulation of specialised (secondary) metabolism, either directly (through sequence-specific RNA silencing or validated RNA-protein interactions affecting pathway transcripts) or indirectly (through organellar RNA metabolism, stress-responsive translational reprogramming, or transcript turnover that influences precursor supply and defence programmes). For each module, the table summarises the species, subcellular compartment, tissue/organ context, principal RNA targets or interaction partners, and the reported link to specialised metabolism. Evidence is classified as mechanistic when it comprises direct RNA binding (or defined RNA-RNA pairing) together with functional perturbation producing concordant effects on RNA fate and/or metabolite output. Associative evidence includes co-localisation, enrichment, correlation, or prediction without causal validation (Table 1).

Abbreviations: AGO, Argonaute; RISC, RNA-induced silencing complex; DCL1, Dicer-like 1; HYL1/DRB1, Hyponastic Leaves 1/Double-stranded RNA-Binding Protein 1; SGs, stress granules; PPR, pentatricopeptide repeat; ATtRACT, A database of RNA-binding proteins and associated motifs; RBPDB, RNA-Binding Protein DataBase; POSTAR3, Post-transcriptional Regulation Database 3; oRNAment, a database of putative RBP target sites/motifs.

Stress granules (SGs) constitute an additional category of RNP [18]. Under stress, SGs can transiently enrich translationally repressed mRNPs. SGs are dynamic cytoplasmic assemblies that form in response to adverse conditions such as elevated temperature, desiccation, oxidative stress, or pathogen invasion. They can act as repositories for untranslated mRNAs, preserving selected transcripts during stress while enabling synthesis of key stress-response proteins [19]. This reprogramming may conserve energy and prioritise survival processes, including the production of specialised metabolites [20].

Processing bodies (P-bodies) and SGs are cytoplasmic granules implicated in mRNA sequestration, storage, and decay. P-bodies act as regulatory hubs that influence mRNA stability and degradation rates, thereby affecting the availability of transcripts encoding enzymes or regulatory proteins involved in metabolite formation [21]. The functional interplay between SGs and P-bodies indicates that plants can adjust protein synthesis in response to diverse internal and external cues [22]. P-bodies promote deadenylation/decapping and exonucleolytic decay, which can limit accumulation of transcripts that are not required under prevailing conditions and enable resource reallocation [22].

Non-coding RNA–protein complexes represent a further class of RNPs alongside mRNPs, SGs, and P-bodies. These complexes involve small interfering RNAs (siRNAs), microRNAs (miRNAs), and long non-coding RNAs (lncRNAs) and mediate RNA silencing and gene regulation [23]. They can modulate post-transcriptional gene activity through RNA cleavage, translational repression, and changes in transcript abundance [24]. In the context of secondary metabolism, such complexes influence the expression of genes and transcription factors associated with metabolite production [25].

RNA-protein complexes orchestrate multiple post-transcriptional processes [27]. Spliceosomal RNPs mediate pre-mRNA splicing by removing non-coding segments and joining coding sequences [28]. This processing generates functional transcripts and can contribute to proteome diversity, with downstream implications for metabolism [28]. After splicing, mRNAs are packaged into distinct mRNPs and exported to the cytoplasm, where further remodelling can contribute to subcellular targeting, including localisation near biosynthetic sites such as plastids or the endoplasmic reticulum [29]. Associated RNP factors regulate protein synthesis from these mRNAs in accordance with developmental stage, tissue type, or stress condition [29]. In parallel, P-body-mediated decay pathways remove unnecessary RNA signals, thereby limiting redundant biosynthesis and conserving cellular resources [30].

RNPs contribute to secondary metabolite synthesis by governing when and where biosynthetic enzymes are expressed and, consequently, how much enzyme is produced [27]. Through control of mRNA stability, localisation, and translation, RNPs influence the duration and abundance of transcripts used for metabolite synthesis, aligning production with environmental and developmental cues [28]. During pathogen challenge, SG-associated translational reprogramming may reduce global translation while enabling production of defence-related enzymes and metabolites, including phytoalexins and flavonoids [29]. Conversely, P-body pathways may promote degradation of mRNAs that constrain synthesis of particular compounds, thereby increasing their abundance during stress [30].

Overall, RNA-protein complexes do not merely transport genetic information; they actively regulate post-transcriptional gene expression and are integral to plant cell function [30]. Their structural diversity and dynamic behaviour position them as integrative platforms that respond to environmental change and support metabolic adjustment. Understanding RNP formation, activity, and regulation in the context of secondary metabolite biosynthesis may inform more targeted modulation of metabolic pathways to enhance plant resilience and phytochemical production [29,30].

Plants rely extensively on RNA-binding proteins (RBPs) to regulate gene expression after transcription [30]. RBPs modulate RNA function and influence diverse physiological processes, including secondary metabolism (Table 2). They regulate gene expression post-transcriptionally by binding specific RNA sequences or structures (often in untranslated regions (UTRs)) and thereby influencing splicing, stability, localisation, and translation. In specialised metabolism, RBPs can, in principle, modulate pathway output by controlling transcripts encoding: (i) biosynthetic enzymes (e.g., CHS/CHI/F3H/DFR in flavonoids; terpene synthases; CYPs); or (ii) pathway regulators (transcription factors (TFs) and signalling nodes) [31]. In this context, RBPs act as precision regulators that shape mRNA splicing, stability, localisation, and translational efficiency, ensuring that biosynthetic enzymes are produced at the appropriate time, location, and abundance in response to developmental cues and environmental signals [32].

**Table 2:** Plant RBP classes potentially relevant to specialised metabolism.

RBP class	Key domains	Species examples	Compartment	Likely RNA-level function	Specialised-metabolism relevance	Evidence type	Citation/Evidence
PUF/Pumilio (APUM)	PUF repeats	*Arabidopsis*	Cytoplasm	Sequence-specific binding in 3′UTRs; translation/decay control	Candidate regulators of stress-responsive programs that can include specialised metabolism (often indirect)	Mostly associative for metabolism-specific claims	APUM stress/regulatory context [[30], [31], [32], [33], [34], [35]]
Glycine-rich RBPs	RRM + Gly-rich	Crops/*Arabidopsis*	Nucleus/cytoplasm	Stress-responsive RNA stabilisation/processing	Likely affects stress reprogramming; specialised-metabolism links often indirect	Associative unless target RNAs validated	Plant SG/P-body and stress-RNA granules context [[36], [37], [38]]
dsRNA-binding proteins (DRBs)	dsRBD	*Arabidopsis*	Nucleus/cytoplasm	miRNA/siRNA biogenesis with DCLs	Enables miRNA networks that regulate flavonoid/phenylpropanoid pathways	Mechanistic for small RNA pathways	DRB/DCL specificity [[37], [38], [39], [40]]
Organelle RBPs (incl. PPR)	PPR motifs	Plants broadly	Chloroplast/mitochondria	RNA editing/splicing/stability	Indirect effects via plastid function/precursor/redox	Mechanistic for RNA metabolism; indirect for specialised metabolism	Organelle RNA metabolism review [[36], [37], [38], [39], [40]]

This table summarises major classes of plant RBPs that may influence specialised (secondary) metabolic pathways. For each class, the table reports the defining RNA-binding domains, representative species, predominant subcellular localisation, and the primary RNA-level functions (e.g., sequence-specific binding in untranslated regions, RNA stabilisation, processing, translational control, or roles in small-RNA biogenesis). The final columns describe proposed links to specialised metabolism, distinguishing direct effects on pathway-relevant transcripts from indirect effects mediated through broader stress programmes, organellar function, redox balance, or precursor supply. Evidence is classified as mechanistic where a defined role in RNA metabolism has been validated (e.g., miRNA/siRNA biogenesis) and as associative where relevance to specialised metabolism is inferred from stress responsiveness, co-expression, localisation, enrichment, or prediction in the absence of validated targets or metabolite readouts (Table 2).

Abbreviations: RBP, RNA-binding protein; PUF, Pumilio/FBF; APUM, *Arabidopsis thaliana* Pumilio; UTR, untranslated region; RRM, RNA recognition motif; Gly-rich, glycine-rich; dsRNA, double-stranded RNA; DRB, double-stranded RNA-binding protein; dsRBD, double-stranded RNA-binding domain; DCL, Dicer-like; siRNA, small interfering RNA; miRNA, microRNA; PPR, pentatricopeptide repeat.

Among the best supported RNP-linked mechanisms affecting specialised metabolism is microRNA (miRNA)-guided regulation executed by AGO-containing RNA-induced silencing complexes (RISC). Multiple studies synthesise evidence that miRNAs regulate TF networks controlling flavonoid/phenylpropanoid biosynthesis and stress-induced remodelling of these pathways [32]. Here, the RNP (AGO plus small RNA) provides a direct post-transcriptional mechanism via cleavage or translational repression that can rapidly adjust enzyme and regulator abundance [31].

Plant RBPs are structurally and functionally diverse, with multiple classes defined by conserved RNA-binding domains [33]. Pumilio and FBF (PUF) proteins are widely studied; they typically recognise sequence motifs in the 3′ UTRs of target mRNAs and have been associated with translational repression or activation [32,33]. Their modularity and sequence specificity enable fine-tuning of transcripts relevant to secondary metabolite biosynthesis. Another major group comprises glycine-rich RBPs, which contain glycine-rich motifs together with RNA recognition motifs (RRMs) or zinc finger domains [34]. These RBPs are often induced by abiotic stress and have been linked to stress-associated metabolic reprogramming, including activation of antioxidant flavonoid pathways.

Cold shock domain proteins, originally described in bacteria, are also present in plants and bind single-stranded RNA, limiting formation of deleterious structures during temperature fluctuations [36]. Their proposed capacity to stabilise transcripts encoding biosynthetic enzymes suggests a link between temperature stress responses and dynamic regulation of secondary metabolite levels, consistent with cold-induced anthocyanin accumulation [36]. In addition, proteins harbouring KH (K-homology) and RRM domains are among the most common RNA-binding proteins in eukaryotes. These domains enable RBPs to bind specific RNA sequences or structures in UTRs (e.g., AU-rich regions, hairpins, and stem-loop elements), thereby influencing transcript decay and translation according to cellular demands [37].

The mechanistic effects of RBPs in secondary metabolism derive from their ability to recognise and bind specific RNA motifs within biosynthetic mRNAs [38]. Such motifs are frequently located in UTRs, which serve as regulatory hotspots for post-transcriptional control. RBPs can protect transcripts from exonucleases or promote decay by recruiting degradation machinery in processing bodies [39]. By interacting with ribosome recruitment sites, RBPs also modulate translational efficiency by promoting or inhibiting translation initiation under specific conditions [40]. This responsiveness allows plants to shift metabolism without the delays associated with transcriptional reprogramming, which is particularly relevant under stress when defensive compounds must be produced rapidly.

Case studies from medicinal plants provide evidence for the involvement of RBPs in secondary metabolism [[41], [42], [43]]. Abiotic and biotic stresses (and elicitors such as jasmonates) strongly reprogramme artemisinin and terpenoid indole alkaloid (TIA) pathway gene expression; however, direct mechanistic mapping of specific RBPs binding specific pathway mRNAs in these medicinal plants remains limited [42,43]. *Artemisia* and *Catharanthus* are high-value systems for specialised metabolism; nevertheless, without direct RNA-protein binding evidence and functional perturbation (e.g., loss- or gain-of-function with concordant RNA fate and metabolite changes), proposed roles for RBPs should be framed as evidence gaps and treated as hypothesis-generating rather than causal. This represents a priority area for CLIP/RIP-seq and functional perturbation studies.

Specific RBPs are thought to regulate mRNA stability and translation for enzymes such as amorpha-4,11-diene synthase (ADS) and cytochrome P450 oxidases required for artemisinin synthesis [43]. In *Catharanthus roseus*, a model system for studying TIA biosynthesis, RBPs have been reported to regulate strictosidine synthase and other key enzymes [43]. RBPs may also contribute to targeting TIA pathway transcripts to subcellular sites where the requisite reactions occur, thereby increasing production efficiency [44]. Together, these examples indicate increasing recognition of RBPs as regulators within the post-transcriptional environment of secondary metabolism [45].

Beyond their endogenous functions, RBPs have been proposed as targets for metabolic engineering [40]. Modulating specific RBPs, or their RNA interactions, may offer a route to more precise control of genes that generate high-value compounds than approaches that rely solely on transcriptional activation or repression [41]. As omics technologies and RNA interactome profiling advance, functional dissection of RBPs across plant species may reveal additional regulatory circuits and inform strategies to increase the production of high-value secondary metabolites in medicinal and agricultural crops [42].

### 2.2 Biophysical properties of RNA-protein complexes

RNA-protein complexes (RNPs) regulate RNA metabolism and possess physicochemical properties that shape their contributions to gene regulation, including in plant secondary metabolism [45]. Many RNPs form membraneless condensates via liquid-liquid phase separation (LLPS), enabling reversible compartmentalisation of RNA metabolic processes during stress. In plants, stress granules (SGs) and processing bodies (P-bodies) are major LLPS-associated assemblies that govern translational repression, RNA storage, and RNA decay [46]. Plant-focused reviews describe dynamic interactions among polysomes, SGs, and P-bodies and outline how these interactions reprioritise cellular activities under stress. However, links between condensate dynamics and specialised-metabolite outputs are often associative unless studies quantify both (i) granule/RNA partitioning and (ii) metabolite changes under defined perturbations [47]. During stress when metabolic reprogramming and energy conservation are critical these condensates provide a potential route to modulate post-transcriptional processes [47].

Through LLPS, RNP granules can act as hubs for translational control and transcript sequestration [48]. Plant cells rapidly remodel translation in response to abiotic stresses (e.g., drought, salinity, heat) and pathogen challenge [49]. This sequestration can suppress translation while protecting transcripts that encode biosynthetic enzymes for metabolites such as flavonoids, alkaloids, and terpenoids. When stress abates, these granules can break down, allowing stored mRNAs to re-enter the translational pool. By contrast, P-bodies are more closely associated with RNA degradation and turnover, facilitating removal of superfluous or damaged transcripts [50]. Collectively, these granules act as spatiotemporal regulators of mRNA fate, enabling context-dependent control of biosynthetic gene expression.

RNP granule assembly and behaviour during LLPS are governed by distinct molecular mechanisms [51]. Intrinsically disordered regions (IDRs) within RNA-binding proteins (RBPs) are particularly important in this context [51]. Because IDRs lack a fixed three-dimensional structure, they can engage in diverse, transient interactions with proteins and RNAs [52], supporting the fluid and reversible properties of RNP granules. IDRs often contain low-complexity sequences enriched in polar and charged residues, which promote the weak, non-covalent interactions required for phase separation [51]. This structural plasticity may enable RBPs to respond to changes in cellular conditions (e.g., ion concentration, pH, oxidative state) that are commonly perturbed during biotic and abiotic stress [53].

RNA also contributes substantially to RNP biophysics. RNA secondary structures including stem-loops, bulges, and hairpins can influence granule formation, specificity, and stability [13]. Such structures can provide scaffolds for recruiting RBPs and additional RNAs, with sequence- and structure-specific interactions shaping granule composition and function [54]. For example, enrichment of mRNAs carrying stress-responsive elements or G-quadruplex motifs may preferentially target them to granules, thereby modulating the availability of transcripts implicated in secondary metabolite production in response to environmental cues [55]. These coupled RNA-protein determinants support a flexible post-transcriptional regulatory network.

RNP granules may therefore contribute to regulation of metabolic genes, particularly within secondary metabolism, where synthesis of bioactive compounds must be tightly coordinated with developmental and environmental signals [55]. Under stress, RNP-mediated sequestration or stabilisation of mRNAs encoding key biosynthetic enzymes may buffer metabolic output by suppressing energy-intensive secondary metabolism when resources are limited, or by enabling increased production when defence or adaptation is required. For instance, during pathogen infection, phase-separated RNP granules may transiently sequester or stabilise transcripts encoding phytoalexin biosynthetic enzymes, supporting timely increases in antimicrobial compound synthesis [13,56]. Conversely, under prolonged abiotic stress, these granules may suppress specific metabolic pathways to conserve energy. Thus, the biophysical properties of RNPs particularly their capacity to form dynamic membraneless organelles via LLPS represent an additional layer of post-transcriptional regulation in plants [56]. This regulation integrates RBP and RNA structural features with physiological cues to balance transcript availability, translation, and decay. Such flexibility is relevant to RNA homeostasis and to optimisation of secondary metabolite production under changing environmental conditions. Understanding the molecular ‘grammar’ and phase behaviour of plant RNPs may enable targeted modulation of metabolic pathways and stress resilience in agricultural and medicinal plants.

### 2.3 Spatial and temporal regulation of RNPs in metabolite biosynthesis

RNA-protein complexes (RNPs) involved in plant secondary metabolism are regulated through spatial and temporal processes that govern their assembly, function, and disassembly [55,56]. Subcellular localisation is central to this regulation because it influences the fate of RNA transcripts implicated in metabolite production (Table 3) [57]. In plant cells, RNPs localise to multiple compartments, including the cytoplasm, nucleus, and organelles such as chloroplasts and mitochondria, each contributing to post-transcriptional control of gene expression [58]. Messenger ribonucleoproteins (mRNPs), stress granules (SGs), and processing bodies (P-bodies) act as cytoplasmic hubs for RNA storage, degradation, and translational repression [20,59]. These structures are particularly important under stress, when they selectively regulate translation of biosynthetic mRNAs to align metabolite output with physiological requirements. In the nucleus, RNPs mediate RNA splicing, modification, and export, ensuring that transcripts encoding enzymes in secondary metabolite pathways are correctly processed and delivered to the cytoplasm [38,60]. Some nuclear RNA-binding proteins (RBPs) interact with non-coding RNAs and chromatin modifiers, providing an interface between transcriptional and post-transcriptional regulation [61]. Chloroplast-localised RNPs, although most often studied in relation to photosynthetic mRNAs, have also been linked to regulation of plastidial pathways that intersect with specialised metabolism, including synthesis of isoprenoids and carotenoids [58,62]. Together, these distributions support compartment-specific roles for RNPs in controlling information flow and maintaining metabolic fidelity.

**Table 3:** Spatial and temporal regulation of RNA-Protein complexes in Plant secondary metabolite biosynthesis.

Dimension	Features	Mechanisms/Examples	Functional Implications	Citation/evidence (representative)
**Subcellular localisation**	Cytoplasmic compartments (stress granules, P-bodies, mRNPs)	SGs sequester translationally repressed mRNAs; P-bodies concentrate decapping/5′→3′ decay factors (e.g., DCPs/XRN) and enable storage vs decay decisions	Rapid reprioritization of enzyme production under stress; preserves capacity for fast re-initiation of specialised-metabolite biosynthesis	Live-cell imaging + polysome profiling + granule proteomics; functional genetics in granule components [55,56]
	Nuclear compartments (speckles/spliceosome-linked RNPs)	Alternative splicing control, splice-regulator relocalization, mRNP maturation/export competency	Ensures correct isoforms/abundance of biosynthetic enzymes and upstream regulators; prevents “mis-assembled” pathway outputs	Nuclear speckle localisation studies + splice-factor perturbation + isoform-resolved RNA-seq [[57], [58], [59], [60], [61], [62]]
	Organelle-localized RNPs (chloroplasts/mitochondria)	PPR-rich RNPs mediate organellar RNA stabilisation, editing, splicing, translation—impacting plastid/mitochondrial functions tied to terpenoid/isoprenoid precursors	Couples photosynthesis/respiration status to precursor supply and downstream specialised metabolism	Reverse genetics + organelle transcript/translation assays; extensive PPR functional literature [[62], [63], [64], [65]]
**Tissue/organ specificity**	Leaf, root, flower, fruit	Tissue-biased expression of RBPs/RNP modules aligns with organ-specific transcriptomes	Matches metabolite profiles to organ roles (defense, attraction, storage)	Spatiotemporal transcriptomics/single-cell or single-nucleus atlases supporting spatial restriction of pathway programs [[65], [66], [67], [68], [69], [70]]
	Specialised cell types (trichomes, laticifers, epidermis)	Glandular trichomes act as dedicated “biofactories” for compounds (e.g., artemisinin); laticifers compartmentalize defense chemistry	Spatially restricted, high-flux biosynthesis while limiting autotoxicity and enabling targeted defense	Tissue/cell-type mapping + cell-specific expression and functional studies in trichome systems; laticifer-focused metabolite/defense reviews [[60], [61], [62], [63], [64]]
**Developmental stage specificity**	Seedling → juvenile → mature → senescent	Developmentally tuned formation/function of RNA granules and decay machinery (translation↔storage↔decay shifts)	Synchronizes specialised metabolism with growth phase (resource allocation, defense onset, senescence remobilization)	Developmental genetics in decapping/P-body factors; RNA granule dynamics reviews [[55], [56], [57], [58]]
	Differentiation/hormonal programs (JA/GA/others)	Signaling cues modulate RNP composition/condensate behavior (often via rapid post-translational regulation of RBPs)	Prioritizes defense vs development-linked metabolites consistent with ontogenic needs	LLPS/condensate frameworks linking environmental + hormonal cues to gene regulation [[59], [60], [61], [62], [63]]
**Environmental responsiveness**	Abiotic stress (heat, drought, salinity, cold, oxidative)	Stress-triggered SG assembly sequesters mRNAs and suppresses translation; supports survival and recovery programs	Saves energy while keeping pathway mRNAs poised for reactivation; can redirect flux to protective metabolites	Stress granule assembly/behavior under abiotic stress; RBP-centric SG regulation reviews [[61], [62], [63], [64]]
	Light/temperature modulation	Light/temperature-responsive nuclear condensates (e.g., phyB photobodies via LLPS) act as regulatory microreactors	Aligns specialised metabolite outputs (e.g., flavonoid/anthocyanin-linked programs) with light/thermal environment	Condensate/photobody LLPS evidence and functional reviews [[60], [61], [62], [63]]
	Biotic stress (pathogen/herbivory)	RNA decay machinery (e.g., decapping complex) can modulate immune outputs; small-RNA regulation can shift phenylpropanoid defenses	Boosts antimicrobial metabolite programs and immune readiness	Mechanistic signaling-to-decapping links + functional immunity studies; small-RNA phenylpropanoid regulation in immunity [[64], [65], [66]]
**Molecular/biophysical features**	IDRs in RBPs	Weak multivalent interactions promote condensate formation and fast remodelling	Enables rapid on/off switching of transcript fate and pathway enzyme production	Plant RBP phase-separation syntheses; condensate/IDR frameworks [[56], [57], [58], [59], [60], [61], [62], [63]]
	RNA secondary structures (stem-loops, G-quadruplexes, bulges)	Structured motifs recruit specific RBPs and influence translation/splicing/stability	Adds selectivity (which biosynthetic mRNAs are targeted, when, and where)	Plant RNA G-quadruplex/structure reviews; structure-function evidence [[52], [53], [54]]
	Post-translational modifications (phosphorylation/redox/others)	PTMs alter RBP affinity, decapping/decay activity, and condensate dynamics (e.g., kinase inputs to P-body factors)	Fast stress/hormone integration without de novo transcription	Demonstrated kinase-to-decapping regulation in Arabidopsis; broader LLPS cue integration [[51], [52], [53], [54], [55]]
**Functional consequences**	Assembly/disassembly dynamics	LLPS enables reversible sequestration and context-dependent processing	Protects transcripts or enforces silencing/decay depending on metabolic priorities	Condensate biology in plants (cytoplasmic + nuclear) [[56], [57], [58], [59], [60], [61], [62], [63], [64]]
	Signal integration	Cross-talk among light/temperature/stress/hormones channels through condensate state changes	Harmonizes environmental + developmental inputs with post-transcriptional control of pathway genes	Light/temperature LLPS photobody framework; LLPS gene-expression integration reviews
	Translation vs degradation control	Shuttling of mRNAs between polysomes, SGs, and P-bodies determines protein output	Optimizes enzyme supply while balancing energetic cost and defense readiness	Polysome-SG-P-body interplay syntheses; decapping genetics [[65], [66], [67], [68], [69], [70], [71]]

This table summarises how RNP localisation, dynamics and biophysical properties shape post-transcriptional control of specialised-metabolism transcripts across cellular, tissue, developmental and environmental contexts. Dimensions include subcellular localisation (cytoplasmic messenger RNPs (mRNPs), stress granules and processing bodies; nuclear speckle/spliceosome-associated RNPs; chloroplast- and mitochondrion-localised RNPs), tissue and cell-type specificity (organ- and cell-restricted expression of RBPs/RNP modules), developmental stage and hormonal programmes, and environmental responsiveness (abiotic and biotic stresses; light and temperature signalling). The table also highlights molecular and biophysical features including intrinsically disordered regions in RBPs, RNA secondary-structure motifs and post-translational modifications-that regulate condensate assembly/disassembly and RNA fate decisions. For each dimension, representative mechanisms and examples are linked to functional consequences for specialised-metabolite biosynthesis, including rapid translational reprioritization, transcript storage versus decay, and coupling of organellar status to precursor supply (Table 3).

Abbreviations: SGs, stress granules; P-bodies, processing bodies; mRNPs, messenger ribonucleoprotein complexes; DCPs, decapping proteins; XRN, 5′→3′ exoribonuclease; PPR, pentatricopeptide repeat; RNPs, ribonucleoprotein complexes; RBPs, RNA-binding proteins; LLPS, liquid-liquid phase separation; IDRs, intrinsically disordered regions; PTMs, post-translational modifications; JA, jasmonic acid; GA, gibberellic acid.

Beyond subcellular localisation, RNP activity is tissue-specific and developmentally regulated, further expanding its regulatory scope [63]. The abundance and function of RBPs and associated RNP granules vary across organs (e.g., leaves, roots, flowers, and fruits), consistent with organ-specific metabolic profiles [64]. In medicinal plants such as *Catharanthus roseus*, alkaloid production is closely coupled to developmental regulation of gene expression in specific cell types, including laticifers and epidermal cells [65]. In this context, RNPs may selectively stabilise or silence transcripts encoding rate-limiting enzymes, thereby coordinating metabolite production with developmental cues. Similarly, in *Artemisia annua*, which produces the antimalarial compound artemisinin, developmental processes such as trichome maturation coincide with differential RNP activity that modulates translation of biosynthetic mRNAs [66]. These stage-specific dynamics are often shaped by hormonal signalling networks, including jasmonates and gibberellins, which interact with RNP machinery to prioritise particular metabolic outputs [67].

In addition to developmental control, RNP dynamics are highly sensitive to environmental stressors [13]. Light, temperature, drought, and pathogen attack can induce assembly or remodelling of RNP granules, enabling plants to adjust metabolic priorities in real time. For example, light-regulated RNPs have been reported to influence translation of photosynthesis-related genes and, indirectly, production of light-induced secondary metabolites such as flavonoids and anthocyanins [68]. Under temperature stress or drought, SGs rapidly sequester transcripts involved in primary and secondary metabolism, potentially buffering against energy depletion and oxidative damage [20]. During biotic stress, such as pathogen infection, immune-associated RNPs may stabilise transcripts encoding phytoalexin biosynthetic enzymes, supporting rapid and localised synthesis of antimicrobial compounds.

RNPs can function as sensors and transducers of environmental signals through dynamic assembly and interactions with signalling molecules [69]. Many RBPs contain regulatory domains that respond to redox changes, phosphorylation, or secondary-messenger binding, which can alter RNA-binding affinity and phase behaviour [70]. Such post-translational modifications enable rapid reprogramming of RNP composition and function in response to external cues, linking metabolic defence strategies with cellular homeostasis [71].

Overall, spatiotemporal regulation of RNPs provides a mechanism for directing metabolite production in plants. By integrating compartmental localisation, developmental specialisation, and environmental responsiveness, RNPs act as nodes within multilayered regulatory networks that govern synthesis of diverse secondary metabolites. Understanding these dynamics may refine interpretations of plant adaptive physiology and inform metabolic engineering strategies for improving production of high-value phytochemicals in agricultural and medicinal crops.

### 2.4 Interplay between noncoding RNAs and RNA-binding proteins (RBPs)

Interactions between noncoding RNAs (ncRNAs) and RNA-binding proteins (RBPs) constitute an important regulatory mechanism in plant gene expression, including in secondary metabolism [21]. Unlike messenger RNAs (mRNAs), ncRNAs do not encode proteins (Table 4). Major classes include long noncoding RNAs (lncRNAs), microRNAs (miRNAs), and circular RNAs (circRNAs). Instead of serving as templates for translation, ncRNAs can act as molecular scaffolds, decoys, or sponges that modulate RBP availability and activity [72]. Through sequence- and structure-dependent interactions, ncRNAs influence the stability, localisation, and translational efficiency of target mRNAs in specialised metabolic pathways [31].

**Table 4:** ncRNA-RBP interactions shaping plant secondary metabolism.

Molecular component	Key structural features	RBP partner(s)/interaction mode	Regulatory effect on secondary metabolism	Example/condition	Citation/evidence (assays; strength)
lncRNA (scaffold/bait for RBPs)	>200 nt; modular domains enabling multivalent binding	lncRNA recruits or retains RBPs to form lncRNA-RBP complexes that tune translation/stability of downstream mRNAs	Indirect to direct (emerging): post-transcriptional gating of pathway TFs/enzymes; strongest evidence currently comes from stress/development models, with increasing relevance to metabolite programs	CRIR1-MeCSP5 in *Manihot esculenta* (cassava) under cold stress: lncRNA binds a cold-shock domain protein (RBP) and increases its translational yield	Mechanistic lncRNA-RBP interaction plus functional genetics reported for CRIR1-MeCSP5; broader plant lncRNA functional frameworks [[72], [73], [74]]
lncRNA (decoy/sink for RBPs)	Structured motifs mimicking RBP targets	lncRNA sequesters RBPs away from cognate mRNA targets (competitive binding)	Putative: may suppress or retime translation of metabolite-related mRNAs by limiting RBP availability; few secondary-metabolism-specific validations so far	Conceptual mode widely discussed for plant lncRNAs; secondary-metabolism examples remain limited	Mechanism class summarized in plant lncRNA reviews; largely review-level evidence, with case-by-case validation still needed [[73], [74], [75]]
miRNA (AGO-RISC module)	21-24 nt guide RNA produced from hairpin precursors	miRNA is loaded into Argonaute (AGO), an RBP, to form RISC and guide cleavage or translational inhibition of targets	Direct: represses pathway TFs/enzymes to fine-tune flavonoids, phenylpropanoids, terpenoids, and alkaloids	miR858 → MYB targets → flavonoid regulation in *Arabidopsis* under immunity/stress contexts	Primary studies linking miR858 perturbation to flavonoid shifts, supported by secondary-metabolism miRNA reviews [[74], [75], [76], [77]]
RBP-mediated control of miRNA activity (sequestration/competition)	Protein-centric; depends on AGO partitioning/localisation and may be tissue specific	RBPs, notably AGO paralogs, compete for miRNAs or spatially sequester them, reshaping where and when silencing occurs	Indirect but mechanistic: this principle can gate miRNA repression of metabolite genes in specific cells or times; pathway-specific demonstrations are still emerging	AGO10 sequesters miR165/166 relative to AGO1 in *Arabidopsis*; exemplar of spatiotemporal regulation	Competitive miRNA binding/sequestration by AGO10 is experimentally demonstrated; supported by miRNA-RBP regulatory reviews [[72], [73], [74]]
circular RNAs (circRNAs)	Covalently closed loops; stable and often structured	Proposed to act as RBP sponges and/or scaffolds for RNP assembly	Mostly putative in plants: may alter availability of RBPs that regulate pathway mRNAs; evidence is clearer in global circRNA biology than in pathway-specific metabolite control	Tissue- and stress-specific circRNA landscapes are reported, but pathway-directed cases remain sparse	Plant circRNA mechanism reviews and evidence summaries; resource-scale cataloging (e.g., PlantCircRNA) [[73], [74], [75], [76]]
ncRNA-RBP hubs (multi-motif regulatory RNAs)	Multiple RNA motifs; modular interaction surfaces	One ncRNA recruits multiple RBPs to coordinate processing, localisation, or stability of transcript sets	System-level coordination (emerging): potential synchronization of biosynthetic gene modules, including clustered pathways, at the post-transcriptional level	Framework concept; strongest support comes from mechanistic lncRNA studies and RBP condensate biology rather than metabolite-cluster-specific validation	Plant lncRNA mechanistic syntheses and plant RBP condensate/phase-separation literature [[74], [75], [76], [77]]
Stress-responsive ncRNAs interacting with RBPs	Inducible lncRNAs/circRNAs with stress-tuned expression	Stress-induced ncRNAs engage RBPs involved in RNA fate control (translation/stability and sometimes chromatin-linked complexes)	Often indirect: shifts defense- and stress-metabolite programs by reprogramming post-transcriptional priorities	Stress-ncRNA networks broadly documented; secondary-metabolism framing highlighted in recent syntheses	Perspectives on ncRNA regulation of secondary metabolism and stress/lncRNA syntheses [[78], [79], [80], [81], [82]]

This table summarises non-coding RNA (ncRNA) classes and interaction modes through which ncRNAs engage RNA-binding proteins (RBPs) to influence specialised (secondary) metabolic regulation. For each molecular component, the table reports key structural features, the RBP partner(s) or interaction mechanism (e.g., scaffolding/baiting, competitive decoying, or recruitment into effector complexes), the reported or proposed effects on specialised metabolism, and representative examples or conditions. Evidence strength is indicated in the final column, ranging from mechanistic support (direct ncRNA-RBP interaction demonstrated together with functional perturbation affecting RNA fate and/or metabolite-related outputs) to associative or putative support (inference based on expression patterns, co-localisation, enrichment, correlation, or review-level synthesis without pathway-specific causal validation) (Table 4).

Abbreviations: ncRNA, non-coding RNA; RBP, RNA-binding protein; lncRNA, long non-coding RNA**;** miRNA, microRNA; AGO, Argonaute; RISC, RNA-induced silencing complex; circRNA, circular RNA; RNP, ribonucleoprotein; TF, transcription factor; CRIR1, Cold-Responsive Intergenic lncRNA 1; MeCSP5, *Manihot esculenta* Cold Shock Protein 5.

lncRNAs (typically >200 nucleotides) can provide modular platforms for RBP recruitment, forming ribonucleoprotein complexes that coordinate post-transcriptional regulation [73]. A recurrent motif is the lncRNA-RBP-mRNA tripartite complex, in which a lncRNA binds both an RBP and a target mRNA, thereby facilitating or inhibiting their interaction depending on context [74]. Some lncRNAs may enhance stability of biosynthetic mRNAs by recruiting stabilising RBPs, including glycine-rich or cold-shock domain proteins, which can support sustained enzyme expression within biosynthetic gene clusters [34]. Conversely, other lncRNAs can act as decoys that sequester RBPs away from mRNA targets, thereby constraining metabolic flux through specific pathways [75]. This functional duality highlights the capacity of lncRNAs to shape metabolic gene expression during developmental transitions and environmental stress responses.

miRNAs, typically 21-24 nucleotides in length, are well-established mediators of gene silencing through RNA-induced silencing complexes (RISCs) [76]. Interactions with RBPs can introduce additional regulatory layers beyond canonical mRNA cleavage and translational repression. Some RBPs can buffer miRNA activity by competing for miRNAs or for binding sites within mRNA untranslated regions (UTRs), thereby limiting miRNA access to target transcripts [77]. Such RBP-mediated miRNA buffering may be particularly important in secondary metabolism, where tight control of biosynthetic enzyme expression is required to maintain appropriate metabolite levels [78]. Under stress, RBPs may protect essential biosynthetic transcripts from miRNA-mediated repression, permitting production of protective compounds such as flavonoids, alkaloids, and terpenoids [29].

circRNAs further expand the ncRNA-RBP network. Generated by back-splicing, circRNAs form covalently closed loops that are comparatively stable and are often detected in a tissue- and stress-dependent manner [78]. circRNAs and lncRNAs can function as RBP sponges, diverting RBPs from other RNA targets [78,79]. Their circular structure can also provide multivalent interaction surfaces capable of binding multiple proteins or RNAs simultaneously, enabling roles as hubs in post-transcriptional regulatory circuits [80]. In secondary metabolism, circRNA-RBP interactions have been associated with coordination of transcriptional and post-transcriptional regulation, particularly within biosynthetic gene clusters [29]. These clusters, which are frequently physically co-located in the genome, enable coordinated expression of multiple genes required for metabolite production. ncRNAs may contribute to the spatial and temporal organisation of these clusters by directing RBPs to specific loci or transcripts, supporting coordinated expression and rapid responsiveness to metabolic demands [14].

The functional implications of ncRNA-RBP interactions extend to integration of complex regulatory networks with multiple signalling inputs [14,26]. For example, stress-responsive lncRNAs may bind RBPs involved in chromatin remodelling, linking post-transcriptional regulation with epigenetic memory pathways [81]. Similarly, competition between miRNAs and RBPs can buffer gene expression, supporting stability under transient environmental fluctuations [82]. Such regulatory resilience is likely to be important for sustained production of secondary metabolites implicated in plant defence, pollinator attraction, and allelopathic interactions.

In summary, ncRNA-RBP interactions form a dynamic regulatory layer that shapes expression of biosynthetic gene clusters in plants. By acting as scaffolds, decoys, sponges, or regulatory hubs, ncRNAs modulate RNA processing, stability, and translation via RBPs. This regulatory axis may contribute to the selectivity and flexibility of secondary metabolism and may provide intervention points for metabolic engineering and synthetic biology aimed at increasing phytochemical output in agricultural and medicinal plant species.

### 2.5 Experimental tools to study RNA-protein complexes in plants

Research on RNA-protein complexes (RNPs) in plants has advanced with the development of molecular and biophysical methods that resolve RNA-protein interactions at high resolution [83]. These approaches are essential for elucidating how RNPs regulate gene expression, particularly in secondary metabolite biosynthesis. RNA immunoprecipitation followed by sequencing (RIP-seq) is widely used to identify RNAs bound to a specific RNA-binding protein (RBP) in vivo [84]. In RIP-seq, antibodies against the protein of interest immunoprecipitate RNP complexes from cell lysates, after which co-precipitated RNAs are extracted and sequenced [85]. This approach enables mapping of RBP–RNA interaction networks and supports identification of target transcripts associated with specific metabolic processes [26,31,84].

Crosslinking immunoprecipitation followed by sequencing (CLIP-seq) and related methods (HITS-CLIP, iCLIP, and PAR-CLIP) increase resolution and binding-site specificity [84]. These techniques use ultraviolet (UV) crosslinking to stabilise RNA-protein interactions before immunoprecipitation. Sequencing of crosslinked RNA fragments identifies both the bound transcripts and the precise RNA interaction sites, enabling inference of sequence motifs or secondary structures associated with RBP recognition [84]. CLIP-seq can therefore be used to investigate how particular RBPs shape the post-transcriptional fate of mRNAs encoding enzymes involved in alkaloid, flavonoid, and terpenoid biosynthesis [84].

Chromatin isolation by RNA purification followed by mass spectrometry (ChIRP-MS) is particularly useful for studying long noncoding RNAs (lncRNAs) that act as scaffolds for chromatin-associated RNP complexes [84,85]. Here, biotinylated antisense oligonucleotides hybridise to a target RNA in plant chromatin, enabling isolation of the RNA together with associated proteins for mass spectrometric identification. ChIRP-MS can identify proteins often RBPs or chromatin modifiers linked physically and functionally to metabolically relevant lncRNAs, including those implicated in regulation of biosynthetic gene clusters [86].

In vitro, electrophoretic mobility shift assays (EMSAs) are commonly used to validate direct RNA–protein interactions [87]. This method exploits the slower migration of RNA-protein complexes relative to free RNA in non-denaturing polyacrylamide gels. EMSA supports qualitative confirmation of binding and quantitative estimation of binding affinity, and is therefore useful for assessing the specificity of RBP interactions with metabolite-associated mRNAs or regulatory ncRNAs [84,88]. EMSA is often combined with mutagenesis or competition assays to identify key binding motifs.

Single-molecule fluorescence approaches, including fluorescence resonance energy transfer (FRET) and total internal reflection fluorescence (TIRF) microscopy, provide high spatial and temporal resolution for characterising RNA-protein interactions at the molecular level. These methods enable real-time interrogation of RNP assembly, conformational dynamics, and responses to cellular cues, improving understanding of how RBPs influence RNA fate under physiological and stress conditions [84]. In the context of secondary metabolism, such approaches can help quantify the kinetics of RNP-mediated transcript localisation or translation during rapid shifts in metabolic demand [89].

Despite these advances, RNP research in non-model medicinal plants remains challenging [90]. Many species lack high-quality genomic and transcriptomic resources, limiting development of probes, antibodies, and annotation required for these assays. In addition, low abundance and tissue-specific expression of many secondary-metabolism RNAs and RBPs can constrain experimental feasibility [91]. Genetic toolkits (e.g., CRISPR/Cas systems and stable transformation protocols) that are well established in *Arabidopsis* or rice are often limited or absent in medicinal plants such as *Catharanthus roseus* and *Artemisia annua*, which are key systems for studying alkaloid and terpenoid biosynthesis [92].

Nevertheless, these constraints also motivate methodological innovation. Advances in next-generation sequencing, single-cell transcriptomics, and proteomics can support de novo construction of reference genomes and interactomes in previously under-resourced species [93]. Cross-species antibody reagents, combined with transient expression systems or protoplast assays, can enable functional testing of RNP components in genetically recalcitrant plants [93]. As synthetic biology becomes more integrated with RNP-focused research, heterologous reconstruction of RNP regulatory circuits may help bridge fundamental discovery and metabolic engineering [92,93] Overall, integrating these experimental strategies is important for defining the RNP-based regulatory architecture underlying plant secondary metabolism, with implications for biotechnology, pharmacognosy, and plant stress biology.

### 2.6 Biotechnological and synthetic biology implications

Recent advances in plant molecular biology and synthetic biology have expanded metabolic engineering strategies, with RNPs increasingly viewed as regulatory elements that can be harnessed to modulate gene expression [93]. A central approach is development or repurposing of RBPs to influence metabolite biosynthesis [30]. Identification of secondary metabolism-associated transcripts based on sequence or structure enables RBPs to be engineered to stabilise, destabilise, relocalise, or alter translation of mRNAs encoding biosynthetic enzymes. For example, synthetic RBPs designed to bind untranslated regions (UTRs) of target transcripts can be used to enhance translation of rate-limiting enzymes in alkaloid, terpenoid, and flavonoid pathways [94].

Engineered RNPs can also support RNA-guided localisation or degradation of biosynthetic mRNAs [95]. Fusion of RBPs to RNA transport signals or degradation-promoting motifs can direct key transcripts to subcellular sites where precursor availability is favourable (e.g., plastids or peroxisomes) or promote decay of transcripts whose expression contributes to metabolic bottlenecks or unwanted by-products. This RNA-level spatiotemporal control can add a regulatory layer beyond DNA-targeted editing, enabling tuning of flux through branched metabolic networks without altering genomic sequence [96].

CRISPR-based RNA-targeting tools, including catalytically inactive Cas13 (dCas13), provide an additional route to post-transcriptional regulation [97]. Unlike CRISPR/Cas9, which targets DNA, dCas13 binds specified RNA sequences without cleavage, enabling programmable modulation of RNA fate [98]. Coupling dCas13 to effector domains (e.g., translational repressors, RNA-stabilising factors, or localisation signals) can allow upregulation or downregulation of selected biosynthetic transcripts in developmentally or environmentally responsive ways [97]. These systems can be further refined using inducible promoters or sensor modules that respond to intracellular metabolites or external stressors.

Beyond targeted regulation, RNPs are also being explored as engineering levers in plant ‘factory’ platforms that produce high-value phytochemicals. Such systems often use chassis species such as *Nicotiana benthamiana* and *Arabidopsis thaliana* [99]. Modulation of endogenous or synthetic RNPs can reprogramme post-transcriptional networks that shape metabolite production [75,95]. For example, altering RBPs that repress enzyme-coding transcripts could increase yields, whereas enhancing stabilising RBPs could reduce variability in pathway gene expression under fluctuating environmental conditions [100]. Modular RNP assembly has also been explored in synthetic biology, where designer RBPs combine RNA-recognition and effector modules to build switch-like circuits that activate or repress metabolite production in response to defined molecular cues [101].

Integration of RNP-based control into synthetic biology pipelines supports multi-layer regulation by coupling transcriptional, post-transcriptional, and translational processes to construct robust biosynthetic routes [102]. Synthetic RBPs can coordinate expression across multi-gene pathways by binding shared regulatory regions on multiple transcripts, thereby synchronising translation and increasing overall metabolic capacity [103]. In addition, coupling RNP modules with RNA biosensors or feedback loops can enable dynamic regulation in response to intracellular metabolite levels, supporting homeostatic control of complex pathways [96].

Overall, RNPs offer substantial biotechnological potential for manipulating plant secondary metabolism. Natural RNP functions, together with programmable RBPs, CRISPR/dCas13 platforms, and modular RNA scaffolds, provide avenues for more targeted and context-responsive metabolic engineering. These strategies may be particularly valuable in non-model medicinal plants where conventional genome editing remains difficult. As transcriptome-editing and RNP-characterisation methods mature, RNP-based regulatory network design may expand the capacity to engineer plant biosynthetic systems.

### 2.7 Future perspectives and knowledge gaps

Despite recent advances in understanding post-transcriptional regulation by RNA-protein complexes (RNPs) in plant secondary metabolism, including in medicinal plants, substantial knowledge gaps remain. A major limitation is the restricted functional annotation of RNA-binding proteins (RBPs) in these species. Although simplified model plants such as *Arabidopsis thaliana* have enabled discovery, characterisation, and profiling of RBPs, most medicinal plants (e.g., *Catharanthus roseus*, *Artemisia annua*, *Withania somnifera*) remain underexplored in RBP functional genomics [104,105]. Their complex specialised metabolic pathways often distributed across, or co-regulated by, gene families require systematic definition of RBP repertoires, expression patterns, RNA targets, and biochemical properties. Because comprehensive RBP atlases are lacking for these pharmacologically relevant plants, attempts to modulate metabolite production via post-transcriptional strategies remain sporadic and largely hypothetical.

Progress will require dynamic, high-resolution mapping of RNPs as metabolism responds to developmental programmes, environmental challenges, or elicitor treatments. Secondary metabolite biosynthesis is highly inducible and context-dependent, and transcription often occurs in temporally and spatially patterned bursts within tissues [106]. Accordingly, static snapshots of RNA-protein interactions are unlikely to capture RNP assembly, remodelling, and disassembly during critical periods of metabolic activity [107]. Time-resolved RNA immunoprecipitation sequencing (RIP-seq), photoactivatable ribonucleoside-enhanced crosslinking and immunoprecipitation (PAR-CLIP), and inducible dCas13-based RNA imaging could, in principle, support in situ, real-time characterisation of RNP dynamics in living cells. However, implementing these approaches in medicinal plants will require both methodological innovation and harmonised protocols for tissue sampling, stress or elicitor application, and metabolite profiling. Interdisciplinary integration is therefore central to narrowing these gaps.

Structural biology approaches including cryo-electron microscopy (cryo-EM), nuclear magnetic resonance (NMR) spectroscopy, and X-ray crystallography are important for defining the mechanistic basis of RBP–RNA recognition, phase separation, and effector recruitment [108]. These methods can reveal how subtle changes in protein conformation or RNA secondary structure influence RNP stability and function under changing cellular conditions. Nevertheless, plant structural analyses remain underused, in part because producing and purifying plant RBPs is technically demanding, particularly for proteins containing intrinsically disordered regions (IDRs) and complex post-translational modifications [[109], [110], [111], [112], [113]].

Computational modelling can help bridge structure function gaps. Motif-based prediction of RNA binding, RNA secondary-structure folding, and RBP docking can be integrated with transcriptomic and proteomic datasets to infer regulatory network architectures and prioritise candidate functional RNP associations [114]. Advances in machine learning and artificial intelligence also enable integration of multi-omics data to predict RNP regulators of biosynthetic gene clusters (BGCs) [115]. Such in silico approaches are especially valuable in non-model plants, where experimental resources are limited and genome annotation remains incomplete [115].

Spatiotemporal transcriptomics may further refine understanding of how RNPs coordinate gene expression at cell-type and developmental-stage resolution. Single-cell RNA sequencing, spatial transcriptomics, and multiplexed in situ hybridisation can map biosynthetic transcripts and the RBPs with which they interact at increasingly high resolution [114]. When combined with live-cell imaging and metabolite tracking, these methods may clarify how RNPs control the timing and localisation of metabolite production in response to physiological or environmental stimuli [115]. Future work should therefore prioritise completion of functional RBP catalogues in medicinal plants, development of dynamic profiling of RNA-protein interactions, and integrative frameworks that combine molecular biology, structural analysis, computational modelling, and advanced transcriptomics [116]. These approaches are expected to improve mechanistic understanding of RNP-mediated regulation and inform rational engineering of secondary metabolism in plant systems [115].

Secondary metabolites, including alkaloids, terpenoids, flavonoids, and polyketides, contribute to defence, stress responses, and ecological interactions [116]. Their biosynthesis depends on coordinated regulation of gene expression and enzyme activities. Although transcription factors and epigenetic regulators are widely recognised as key controllers of these pathways, emerging evidence indicates that RNPs add a further regulatory layer by modulating RNA stability, localisation, and translation of biosynthetic genes [117].

RNA-protein interactions involved in secondary metabolism exhibit adaptable biophysical characteristics. Structural motifs such as RNA recognition motifs (RRMs), K homology (KH) domains, and zinc-finger domains can confer high-affinity yet flexible RNA binding [118]. Interfaces within these RBPs are often structurally versatile, enabling recognition of diverse RNA secondary structures, including stem-loops and G-quadruplexes. Binding kinetics are frequently characterised by rapid association and dissociation, supporting responsive regulation to environmental and developmental cues [118]. Many RNA-protein assemblies can also undergo liquid-liquid phase separation to form biomolecular condensates that act as dynamic control hubs for RNA metabolism, integrating signals that rapidly influence secondary metabolite biosynthesis [119].

### 2.8 Functional consequences in metabolite biosynthesis

RNA-protein interactions can have diverse functional consequences for secondary metabolism. These complexes can stabilise mRNAs encoding biosynthetic enzymes or promote their degradation, thereby tuning abundance of key pathway components [115]. RBPs can also repress or activate translation via interactions with untranslated regions of mRNAs encoding enzymes central to metabolic pathways. In addition, RNA-protein complexes can mediate subcellular transport and localisation of mRNAs, supporting synthesis of enzymes in appropriate cellular contexts for multi-step biosynthetic processes [116]. Some RBPs recruit small RNAs or interact with silencing complexes to downregulate competing metabolic branches, further shaping cellular metabolite profiles [117]. Collectively, these mechanisms can support adaptive reconfiguration of secondary metabolism in response to stress, pathogens, and developmental cues.

### 2.9 Comparative and evolutionary perspectives

Comparative studies indicate that RNA-protein regulatory mechanisms associated with secondary metabolism are conserved across diverse kingdoms [111]. In bacteria, riboswitches coupled with RBPs coordinate antibiotic biosynthesis. In fungi, RBPs can stabilise transcripts encoding polyketide synthases and non-ribosomal peptide synthetases, supporting metabolite production [112]. In higher plants, RNA-protein interactions contribute to regulation of flavonoid and alkaloid pathways through post-transcriptional stabilisation and translational modulation of biosynthetic mRNAs [113]. Together, these observations suggest that RNA-protein complexes represent an evolutionarily conserved regulatory framework that complements transcription factor-mediated control of secondary metabolism.

## 3 Conclusion

RNA-protein complexes (RNPs) regulate post-transcriptional gene expression in plants and provide specific, reversible, and context-dependent mechanisms that shape secondary metabolism. Unlike predominantly transcriptional regulatory systems, RNPs modulate mRNA splicing, localisation, stability, and translational efficiency, allowing plants to adjust metabolic output rapidly in response to developmental cues and environmental stress. This flexibility is particularly relevant to secondary metabolite biosynthesis, which is dynamic, spatially restricted, and frequently stress induced. RNPs function as active regulators rather than passive RNA chaperones. They regulate access to the translational machinery, influence the half-life of biosynthetic transcripts, and coordinate subcellular localisation of transcripts at sites of localised gene expression. Their biophysical properties particularly the ability to undergo liquid-liquid phase separation (LLPS) and form membraneless organelles such as stress granules and processing bodies enable rapid, reversible sequestration and release of specific mRNAs as physiological states change. Assembly and disassembly of these condensates depend on intrinsically disordered regions (IDRs) within RNA-binding proteins (RBPs) and specific RNA secondary structures. Through these features, RNPs act as dynamic hubs for transcript sorting and translational control, which is pertinent to secondary metabolism where pathways require tight temporal and spatial regulation to balance growth, defence, and energy allocation. Linking RNP biology with secondary metabolite regulation also suggests opportunities in plant metabolic engineering, stress plasticity, and natural product biosynthesis. Modulating RBP expression, altering RNA-binding patterns, or applying CRISPR/dCas13-based RNA-targeting approaches may enable selective upregulation or repression of specific branches of secondary metabolism without broadly perturbing transcriptional programmes. However, the claim that this approach is ‘far better’ than conventional genetic engineering, and that it minimises off-target effects while maintaining native gene expression profiles, requires supporting evidence. More generally, RNA-level control could permit transient, signal-responsive regulation of metabolite concentrations, which may be valuable for biotechnological applications such as controlled production of pharmacologically active compounds in plant cell cultures or plant-based biofactories. RNP-mediated regulation also informs molecular understanding of plant stress biology. Environmental stresses, including drought, salinity, temperature variation, and pathogen attack, are often associated with rapid reprogramming of secondary metabolism through signalling cascades and RNP dynamics. Overall, RNPs provide a multi-layered regulatory platform that supports flexible and precise control of secondary metabolite biosynthesis in plants through biochemical specificity, biophysical plasticity, and reversibility. Continued research into RNP structure-function relationships, temporal dynamics, and species-specific variation is expected to advance plant systems biology, synthetic biology, and sustainable agriculture, with potential relevance to climate-resilient crop improvement.

## Consent to participate

Not applicable.

## Consent to publish declaration

Not applicable.

## Ethics approval

Not applicable.

## Clinical trial date of registration

Not applicable.

## Clinical trial registration number

Not applicable.

## Clinical trial registry

Not applicable.

## Generative AI disclosure statement

During the preparation of this work, the author(s) used QUILBOT to improve scientific language, clarity, and readability**.** After using this tool, the author(s) reviewed and edited the content as needed and take full responsibility for the content of the published article.

## Funding

None.

## CRediT authorship contribution statement

**Okechukwu Paul-Chima Ugwu:** Conceptualization, Data curation, Formal analysis, Investigation, Methodology, Resources, Supervision, Validation, Visualization, Writing – original draft, Writing – review & editing. **Melvin Nnaemeka Ugwu:** Data curation, Methodology, Supervision, Validation, Visualization, Writing – original draft, Writing – review & editing. **Hope Onohuean:** Conceptualization, Data curation, Methodology, Supervision, Visualization, Writing – original draft, Writing – review & editing. **Hilal Ahmad Rather:** Conceptualization, Data curation, Supervision, Validation, Visualization, Writing – original draft, Writing – review & editing. **Ibe Michael Usman:** Data curation, Methodology, Supervision, Validation, Visualization, Writing – original draft, Writing – review & editing.

## Declaration of competing interest

The authors declare that they have no known competing financial interests or personal relationships that could have appeared to influence the work reported in this paper.

## Data Availability

Data will be made available on request.

## References

[1] Zetzsche J., Fallet M. (2024). To live or let die? Epigenetic adaptations to climate change—a review. Environ. Epigenet..

[2] Aguirre-Becerra H., Vazquez-Hernandez M.C., Saenz de la O.D., Alvarado-Mariana A., Guevara-Gonzalez R.G., Garcia-Trejo J.F., Feregrino-Perez A.A. (2021). Bioactive Natural Products for Pharmaceutical Applications.

[3] Sharma A, Sharma S, Kumar A, Kumar V, Sharma AK. Plant secondary metabolites: an introduction of their chemistry and biological significance with physicochemical aspect. InPlant Secondary Metabolites: Physico-Chemical Properties and Therapeutic Applications 2022 Mar 2 (pp. 1-45). Singapore: Springer Nature Singapore.

[4] Wang M., Zhang S., Li R., Zhao Q. (2024 Dec 24). Unraveling the specialized metabolic pathways in medicinal plant genomes: a review. Front. Plant Sci..

[5] Patil J.R., Mhatre K.J., Yadav K., Yadav L.S., Srivastava S., Nikalje G.C. (2024 Dec). Flavonoids in plant-environment interactions and stress responses. Discover Plants.

[6] Kajla M., Roy A., Singh I.K., Singh A. (2023 Mar 2). Regulation of the regulators: transcription factors controlling biosynthesis of plant secondary metabolites during biotic stresses and their regulation by miRNAs. Front. Plant Sci..

[7] Rabeh K., Hnini M., Oubohssaine M. (2025 Dec). A comprehensive review of transcription factor-mediated regulation of secondary metabolites in plants under environmental stress. Stress Biol..

[8] Dai Z., Ramesh V., Locasale J.W. (2020 Dec). The evolving metabolic landscape of chromatin biology and epigenetics. Nat. Rev. Genet..

[9] Kumar V., Srivastava A.K., AbdElgawad H. (2025 Mar). Transcriptional and post-transcriptional regulation of plant growth, development, and stress responses. J. Plant Growth Regul..

[10] Meraj T.A., Fu J., Raza M.A., Zhu C., Shen Q., Xu D., Wang Q. (2020 Mar 25). Transcriptional factors regulate plant stress responses through mediating secondary metabolism. Genes.

[11] Rissland O.S. (2017 Jan). The organization and regulation of mRNA–protein complexes. Wiley Interdiscip. Rev. RNA.

[12] Marchese D., de Groot N.S., Lorenzo Gotor N., Livi C.M., Tartaglia G.G. (2016 Nov). Advances in the characterization of RNA‐binding proteins. Wiley Interdiscip. Rev. RNA.

[13] Ripin N., Parker R. (2023 Oct 26). Formation, function, and pathology of RNP granules. Cell.

[14] Briata P., Gherzi R. (2020 Sep 17). Long non-coding RNA-ribonucleoprotein networks in the post-transcriptional control of gene expression. Non-coding RNA.

[15] Corley M., Burns M.C., Yeo G.W. (2020 Apr 2). How RNA-binding proteins interact with RNA: molecules and mechanisms. Mol. Cell.

[16] Palazzo A.F., Qiu Y., Kang Y.M. (2024 Dec 31). mRNA nuclear export: how mRNA identity features distinguish functional RNAs from junk transcripts. RNA Biol..

[17] Singh A., Kumar R., Srivastava A. (2024 Jun).

[18] Hodgson R.E. (2019).

[19] Luo Y., Na Z., Slavoff S.A. (2018 Jan 30). P-bodies: composition, properties, and functions. Biochemistry.

[20] Kearly A., Nelson A.D., Skirycz A., Chodasiewicz M. (2024 Mar 15).

[21] Zhang P., Wu W., Chen Q., Chen M. (2019 Sep 25). Non-coding RNAs and their integrated networks. J.Integrative Bioinformatics.

[22] Holoch D., Moazed D. (2015 Feb). RNA-mediated epigenetic regulation of gene expression. Nat. Rev. Genet..

[23] Choquet K., Patop I.L., Churchman L.S. (2025 Apr). The regulation and function of post-transcriptional RNA splicing. Nat. Rev. Genet..

[24] Harvey R., Dezi V., Pizzinga M., Willis A.E. (2017 Aug 15). Post-transcriptional control of gene expression following stress: the role of RNA-binding proteins. Biochem. Soc. Trans..

[25] Al Aboud N.M. (2024). Unlocking the genetic potential: strategies for enhancing secondary metabolite biosynthesis in plants. J. Saudi Soc. Agricul. Sci..

[26] Ruta V., Pagliarini V., Sette C. (2021 Oct 7). Coordination of RNA processing regulation by signal transduction pathways. Biomolecules.

[27] Akbar M.U., Aqeel M., Shah M.S., Jeelani G., Iqbal N., Latif A., Elnour R.O., Hashem M., Alzoubi O.M., Habeeb T., Qasim M. (2023 Oct 1). Molecular regulation of antioxidants and secondary metabolites act in conjunction to defend plants against pathogenic infection. South Afr. J. Bot..

[28] Loll-Krippleber R., Brown G.W. (2017 Sep 15). P-body proteins regulate transcriptional rewiring to promote DNA replication stress resistance. Nat. Commun..

[29] Jiang S., Cui J.L., Li X.K. (2021 Nov 2). MicroRNA-mediated gene regulation of secondary metabolism in plants. Crit. Rev. Plant Sci..

[30] Muleya V., Marondedze C. (2020 Nov 18). Functional roles of RNA-binding proteins in plant signaling. Life.

[31] Fang Y., Liu X., Liu Y., Xu N. (2024 Oct 22). Insights into the mode and mechanism of interactions between RNA and RNA-Binding proteins. Int. J. Mol. Sci..

[32] Nishanth M.J., Simon B. (2020 Jan). Functions, mechanisms and regulation of Pumilio/Puf family RNA binding proteins: a comprehensive review. Mol. Biol. Rep..

[33] Huh S.U. (2021 Dec 9). The role of Pumilio RNA binding protein in plants. Biomolecules.

[34] Cheng K., Zhang C., Lu Y., Li J., Tang H., Ma L., Zhu H. (2023 Oct 9). The glycine-rich RNA-Binding protein is a vital post-transcriptional regulator in crops. Plants.

[35] Zhang Y., Gross C.A. (2021 Nov 23). Cold shock response in bacteria. Annu. Rev. Genet..

[36] Białoskórska M., Rucińska A., Boczkowska M. (2024 Sep 20). Molecular mechanisms underlying freezing tolerance in plants: implications for cryopreservation. Int. J. Mol. Sci..

[37] Laverty KU. *On the RNA-binding Specificities of RNA-binding Proteins* (Doctoral Dissertation, University of Toronto (Canada)).

[38] Georgakopoulos-Soares I., Parada G.E., Hemberg M. (2022 Jan 1). Secondary structures in RNA synthesis, splicing and translation. Comput. Struct. Biotechnol. J..

[39] Nishida K., Kuwano Y., Nishikawa T., Masuda K., Rokutan K. (2017 Jun 23). RNA binding proteins and genome integrity. Int. J. Mol. Sci..

[40] Shirokikh N.E., Preiss T. (2018 Jul). Translation initiation by cap‐dependent ribosome recruitment: recent insights and open questions. Wiley Interdiscip. Rev. RNA.

[41] Vashisth D., Kumar R., Rastogi S., Patel V.K., Kalra A., Gupta M.M., Gupta A.K., Shasany A.K. (2018 Feb 21). Transcriptome changes induced by abiotic stresses in Artemisia annua. Sci. Rep..

[42] Qamar F., Mishra A., Ashrafi K., Saifi M., Dash P.K., Kumar S., Abdin M.Z. (2024 Nov 1). Increased artemisinin production in Artemisia annua L. by co-overexpression of six key biosynthetic enzymes. Int. J. Biol. Macromol..

[43] Liu Y., Patra B., Singh S.K., Paul P., Zhou Y., Li Y., Wang Y., Pattanaik S., Yuan L. (2021 Nov). Terpenoid indole alkaloid biosynthesis in Catharanthus roseus: effects and prospects of environmental factors in metabolic engineering. Biotechnol. Lett..

[44] Biłas R., Szafran K., Hnatuszko-Konka K., Kononowicz A.K. (2016 Nov). Cis-regulatory elements used to control gene expression in plants. Plant Cell Tissue Organ Cult..

[45] Zmudjak M., Ostersetzer-Biran O. (2018 Feb 5). RNA metabolism and transcript regulation. Annu. Plant Rev. Online..

[46] Antifeeva I.A., Fonin A.V., Fefilova A.S., Stepanenko O.V., Povarova O.I., Silonov S.A., Kuznetsova I.M., Uversky V.N., Turoverov K.K. (2022 May). Liquid–liquid phase separation as an organizing principle of intracellular space: overview of the evolution of the cell compartmentalization concept. Cell. Mol. Life Sci..

[47] Ivanov P., Kedersha N., Anderson P. (2019 May 1). Stress granules and processing bodies in translational control. Cold Spring Harbor Perspect. Biol..

[48] Langdon E.M., Gladfelter A.S. (2018 Sep 8). A new lens for RNA localization: liquid-liquid phase separation. Annu. Rev. Microbiol..

[49] Suhorukova A.V., Sobolev D.S., Milovskaya I.G., Fadeev V.S., Goldenkova-Pavlova I.V., Tyurin A.A. (2023 Oct 13). A molecular orchestration of plant translation under abiotic stress. Cells.

[50] Subedi S., Uversky V.N., Tripathi T. (2025 Jan).

[51] Mittag T., Parker R. (2018 Nov 2). Multiple modes of protein–protein interactions promote RNP granule assembly. J. Mol. Biol..

[52] Zeke A., Schad E., Horvath T., Abukhairan R., Szabo B., Tantos A. (2022 Sep). Deep structural insights into RNA‐binding disordered protein regions. Wiley Interdiscip. Rev. RNA.

[53] Damaris R.N., Yang P. (2021). Protein phosphorylation response to abiotic stress in plants. Plant phosphoproteomics: methods and protocols.

[54] Calabretta S., Richard S. (2015 Nov 1). Emerging roles of disordered sequences in RNA-binding proteins. Trends Biochem. Sci..

[55] Hu R., Teng X., Li Y. (2024 Aug 1). Unleashing plant synthetic capacity: navigating regulatory mechanisms for enhanced bioproduction and secondary metabolite discovery. Curr. Opin. Biotechnol..

[56] Fan S., Zhang Y., Zhu S., Shen L. (2024 Feb 27). Plant RNA-binding proteins: Phase-separation dynamics and functional mechanisms underlying plant development and stress responses. Mol. Plant.

[57] Zhang C., Zhu X., Peterson N., Wang J., Wan S. (2025). A comprehensive review on RNA subcellular localization prediction. arXiv preprint arXiv:2504.17162.

[58] Ali N.A., Song W., Huang J., Wu D., Zhao X. (2024 Nov 16). Recent advances and biotechnological applications of RNA metabolism in plant chloroplasts and mitochondria. Crit. Rev. Biotechnol..

[59] Kershaw C.J., Nelson M.G., Lui J., Bates C.P., Jennings M.D., Hubbard S.J., Ashe M.P., Grant C.M. (2021 Nov 12). Integrated multi-omics reveals common properties underlying stress granule and P-body formation. RNA Biol..

[60] Yang S., Kim S.H., Yang E., Kang M., Joo J.Y. (2024 Jun). Molecular insights into regulatory RNAs in the cellular machinery. Exp. Mol. Med..

[61] Sztuba-Solinska J. (2023 Jun 17). Navigating Non-coding RNA: from Biogenesis to Therapeutic Application.

[62] Zanini A.A., Azim M.F., McCray T.N., Burch-Smith T.M. (2024 Nov).

[63] Mangoni D., Mazzetti A., Ansaloni F., Simi A., Tartaglia G.G., Pandolfini L., Gustincich S., Sanges R. (2025 Feb). From the genome's perspective: bearing somatic retrotransposition to leverage the regulatory potential of L1 RNAs. Bioessays.

[64] Sharma B., Prall W., Bhatia G., Gregory B.D. (2023 May 22). The diversity and functions of plant RNA modifications: what we know and where we go from here. Annu. Rev. Plant Biol..

[65] Liu J., Cai J., Wang R., Yang S. (2016 Dec 28). Transcriptional regulation and transport of terpenoid indole alkaloid in Catharanthus roseus: exploration of new research directions. Int. J. Mol. Sci..

[66] Wani K.I., Choudhary S., Zehra A., Naeem M., Weathers P., Aftab T. (2021 Aug). Enhancing artemisinin content in and delivery from Artemisia annua: a review of alternative, classical, and transgenic approaches. Planta.

[67] Rudolf J., Tomovicova L., Panzarova K., Fajkus J., Hejatko J., Skalak J. (2024 Sep 11). Epigenetics and plant hormone dynamics: a functional and methodological perspective. J. Exp. Bot..

[68] Zoschke R., Bock R. (2018 Apr 1). Chloroplast translation: structural and functional organization, operational control, and regulation. Plant Cell.

[69] Ohno H., Saito H. (2016 Jan 1). RNA and RNP as building blocks for nanotechnology and synthetic biology. Prog. Mol. Biol. Transl. Sci..

[70] Jin C., Ye Y., Gao L., Zhong Z., Zhou C., Wu X., Li X., Zhou G., Chen S., Wei Y., Cai L. (2025 Dec). Biological function of RNA-binding proteins in myocardial infarction: a potential emerging therapeutic limelight. Cell Biosci..

[71] Zarnack K., Balasubramanian S., Gantier M.P., Kunetsky V., Kracht M., Schmitz M.L., Sträßer K. (2020 Sep 11). Dynamic mRNP remodeling in response to internal and external stimuli. Biomolecules.

[72] Santosh B., Varshney A., Yadava P.K. (2015 Jan). Non‐coding RNAs: biological functions and applications. Cell Biochem. Funct..

[73] Zhang X., Wang W., Zhu W., Dong J., Cheng Y., Yin Z., Shen F. (2019 Nov 8). Mechanisms and functions of long non-coding RNAs at multiple regulatory levels. Int. J. Mol. Sci..

[74] Janakiraman H., House R.P., Gangaraju V.K., Diehl J.A., Howe P.H., Palanisamy V. (2018 Apr 1). The long (lncRNA) and short (miRNA) of it: tgfβ-mediated control of RNA-binding proteins and noncoding RNAs. Mol. Cancer Res..

[75] Ho J.D., Man J.H., Schatz J.H., Marsden P.A. (2021 Sep). Translational remodeling by RNA‐binding proteins and noncoding RNAs. Wiley Interdiscip. Rev. RNA.

[76] Zhan J., Meyers B.C. (2023 May 22). Plant small RNAs: their biogenesis, regulatory roles, and functions. Annu. Rev. Plant Biol..

[77] Connerty P., Ahadi A., Hutvagner G. (2015 Dec 26). RNA binding proteins in the miRNA pathway. Int. J. Mol. Sci..

[78] Huang A., Zheng H., Wu Z., Chen M., Huang Y. (2020 Feb 10). Circular RNA-protein interactions: functions, mechanisms, and identification. Theranostics.

[79] Kristensen L.S., Andersen M.S., Stagsted L.V., Ebbesen K.K., Hansen T.B., Kjems J. (2019 Nov). The biogenesis, biology and characterization of circular RNAs. Nat. Rev. Genet..

[80] Wang S., Guo B., Wang H., Yang F. (2024 Mar). The optimization strategies of LNP-mRNA formulations: development and challenges for further application. J. Drug Deliv. Sci. Technol..

[81] Yadav A., Mathan J., Dubey A.K., Singh A. (2024 Feb 7). The emerging role of non-coding RNAs (ncRNAs) in plant growth, development, and stress response signaling. Non-coding RNA.

[82] van Wijk N., Zohar K., Linial M. (2022 Dec 18). Challenging cellular homeostasis: spatial and temporal regulation of miRNAs. Int. J. Mol. Sci..

[83] Bheemireddy S., Sandhya S., Srinivasan N., Sowdhamini R. (2022 Oct 7). Computational tools to study RNA-protein complexes. Front. Mol. Biosci..

[84] Yi W., Yan J. (2025). Decoding RNA–Protein interactions: methodological advances and emerging challenges. Adv. Genetics.

[85] Sharma S., Sharma C.M. (2022). Post-Transcriptional Gene Regulation.

[86] Trendel J. *Proteomic Exploration of Protein-RNA Interactions in Human Cells* (Doctoral Dissertation).

[87] He Z., Wang Z., Lu Z., Gao C., Wang Y. (2024 May 12). An electrophoretic mobility shift assay using the protein isolated from host plants. Plant Methods.

[88] Lin C., Miles W.O. (2019 Jun 20). Beyond CLIP: advances and opportunities to measure RBP–RNA and RNA–RNA interactions. Nucleic Acids Res..

[89] Yasuhara T., Xing Y.H., Bauer N.C., Lee L., Dong R., Yadav T., Soberman R.J., Rivera M.N., Zou L. (2022 Aug 4). Condensates induced by transcription inhibition localize active chromatin to nucleoli. Mol. Cell.

[90] Singh D., Mathur S., Prasad M., Ranjan R. (2024 Feb). Next-generation sequencing in medicinal plants: recent progress, opportunities, and challenges. J. Plant Growth Regul..

[91] Singh R., Yadav R., Fatima S.K. (2025 Jan). The engineering of medicinal plants: future prospects and limitations. InMed. Biotechnol..

[92] Inckemann R. Advancing Chloroplast Synthetic Biology by Developing High Throughput Prototyping Capabilities and Novel Genetic Tools.

[93] Garagounis C., Delkis N., Papadopoulou K.K. (2021 Aug). Unraveling the roles of plant specialized metabolites: using synthetic biology to design molecular biosensors. New Phytol..

[94] Burgers L.D., Fürst R. (2021 Aug 1). Natural products as drugs and tools for influencing core processes of eukaryotic mRNA translation. Pharmacol. Res..

[95] Li B., Qu L., Yang J. (2023 Nov 6). RNA-guided RNA modifications: biogenesis, functions, and applications. Accounts Chem. Res..

[96] Zhou S., Alper H.S., Zhou J., Deng Y. (2023 May 19). Intracellular biosensor-based dynamic regulation to manipulate gene expression at the spatiotemporal level. Crit. Rev. Biotechnol..

[97] Yang H., Patel D.J. (2024 Jun). Structures, mechanisms and applications of RNA-centric CRISPR–Cas13. Nat. Chem. Biol..

[98] Tian S., Zhang B., He Y., Sun Z., Li J., Li Y., Yi H., Zhao Y., Zou X., Li Y., Cui H. (2022 Mar 21). CRISPR-iPAS: a novel dCAS13-based method for alternative polyadenylation interference. Nucleic Acids Res..

[99] Singh H. (2024 Jun).

[100] Paris G., Katsuya-Gaviria K., Luisi B.F. (2025 Jan). Machinery, mechanism, and information in post-transcription control of gene expression, from the perspective of unstable RNA. Q. Rev. Biophys..

[101] English M.A., Gayet R.V., Collins J.J. (2021 Jun 20). Designing biological circuits: synthetic biology within the operon model and beyond. Annu. Rev. Biochem..

[102] Gao Y., Wang L., Wang B. (2023 Dec 18). Customizing cellular signal processing by synthetic multi-level regulatory circuits. Nat. Commun..

[103] Zinani O.Q., Keseroğlu K., Özbudak E.M. (2022 Jan 1). Regulatory mechanisms ensuring coordinated expression of functionally related genes. Trends Genet..

[104] Hussain S., Sagar T., Kaur S., Nipunta Kapoor N., Mahajan R. (2023 Aug 12). InBiosynthesis of Bioactive Compounds in Medicinal and Aromatic Plants: Manipulation by Conventional and Biotechnological Approaches.

[105] Halder M., Roy S. (2023 Jul).

[106] Bai Y., Liu X., Baldwin I.T. (2024 Feb 29). Using synthetic biology to understand the function of plant specialized metabolites. Annu. Rev. Plant Biol..

[107] Dai X., Zhang S., Zaleta-Rivera K. (2020 Feb). RNA: interactions drive functionalities. Mol. Biol. Rep..

[108] Renaud J.P. (2020 Feb 5). Structural Biology in Drug Discovery: Methods, Techniques, and Practices.

[109] Hsiao A.S. (2022 May 24). Plant protein disorder: spatial regulation, broad specificity, switch of signaling and physiological status. Front. Plant Sci..

[110] Alum E.U., Uti D.E., Egba S.I., Ugwu O.P., Aja P.M. (2025 Jun). The role of phytochemicals in age-related cognitive decline: a natural solution for brain health. Nat. Prod. Commun..

[111] Alum E.U., Nwuruku O.A., Ugwu O.P., Uti D.E., Alum B.N., Edwin N. (2025 May). Harnessing Nature: Plant-Derived nanocarriers for targeted drug delivery in cancer therapy. Phytomed. Plus.

[112] Okechukwu Paul-Chima U., Chinyere Nkemjika A., Melvin Nnaemeka U., Onohuean H. (2025 Dec 31). Harnessing plant metabolic pathways for innovative diabetes management: unlocking the therapeutic potential of medicinal plants. Plant Signal. Behav..

[113] Paul-Chima Ugwu Okechukwu, Ugwu Chinyere Nneoma, Ugo Alum Esther (October 2024). Integrated approaches in nutraceutical delivery systems: optimizing ADME dynamics for enhanced therapeutic potency and clinical impact. RPS Pharm. Pharmacol. Rep..

[114] Dreyfuss G., Kim V.N., Kataoka N. (2002). Messenger-RNA-binding proteins and the messages they carry. Nat. Rev. Mol. Cell Biol..

[115] Marín M., Ott T. (2012). RNA-binding proteins and their role in the regulation of plant secondary metabolism. Front. Plant Sci..

[116] Lunde B.M., Moore C., Varani G. (2007). RNA-binding proteins: modular design for efficient function. Nat. Rev. Mol. Cell Biol..

[117] Banani S.F., Lee H.O., Hyman A.A., Rosen M.K. (2017). Biomolecular condensates: organizers of cellular biochemistry. Nat. Rev. Mol. Cell Biol..

[118] Bailey-Serres J., Sorenson R., Juntawong P. (2009). Getting the message across: cytoplasmic ribonucleoprotein complexes. Trends Plant Sci..

[119] Romero-Campero F.J., Pérez-Hurtado I., Lucas-Reina E., Romero J.M., Valverde F. (2021). Chimeric RNA–protein complexes and plant secondary metabolism regulation. Plant Cell Environ..

